# Case report and literature review: Acute rhabdomyolysis caused by overheating of electric blanket complicated with Guillain-Barré syndrome

**DOI:** 10.3389/fneur.2024.1362648

**Published:** 2024-02-21

**Authors:** Dongyang Jiang, Ming Zhao, Xiaojun Li, Qiongdan Hu, Qiong Zhang

**Affiliations:** ^1^Department of Nephrology, The Affiliated Traditional Chinese Medicine Hospital, Southwest Medical University, Luzhou, China; ^2^Institute of Integrated Chinese and Western Medicine, Southwest Medical University, Luzhou, Sichuan, China

**Keywords:** rhabdomyolysis, Guillain–Barré syndrome, heatstroke, electric blanket, acute kidney injury, plasma exchange

## Abstract

Rhabdomyolysis (RM) induced by electric blankets is exceedingly rare, with only three cases identified in our literature review. Both RM and Guillain–Barré syndrome (GBS) present with similar clinical manifestations of myalgia and muscle weakness, posing a potential challenge for accurate diagnosis in clinical settings. This report presents the case of a 22-year-old man who developed RM subsequent to the use of an electric blanket. Despite undergoing plasma exchange and renal replacement therapy, the patient continued to exhibit poor muscle strength in both lower limbs. Subsequent comprehensive evaluation revealed the presence of concurrent GBS. Following a 5-day course of intravenous gamma globulin treatment, the patient experienced rapid recovery of muscle strength and was discharged. Additionally, we reviewed seven cases from the literature of coexistent RM and GBS. This indicated that investigation of the timing of onset of muscle strength decline in RM patients could help to identify potential concurrent neurological or muscular disorders. In cases in which concurrent GBS and RM cannot be definitively ascertained during early hospitalization, prioritizing plasma exchange treatment may lead to improved patient outcomes.

## 1 Introduction

The causes of rhabdomyolysis (RM) usually include trauma, drugs, high temperature, strenuous exercise, toxins, and virus infections (1), but acute RM caused by overheating of electric blankets is rarely reported. Guillain–Barré syndrome (GBS) is an immune-mediated polyneurogenic neuropathy with an estimated 100,000 new cases worldwide each year (2). The typical symptom of GBS is rapidly progressive bilateral limb weakness, and some patients initially show muscle or nerve root pain (3), similar to the classic triad of RM (muscle weakness, muscle pain, and myoglobinuria) (4). Diagnosis of RM can be established through clinical manifestations and laboratory tests; however, GBS can be easily overlooked when it coexists with RM. Here, we report a rare case of acute RM caused by overheating of an electric blanket in a patient who also had GBS. Informed written consent was obtained from the patient for the publication of this case report and its associated images and tables.

## 2 Case presentation

We report a 22-year-old male patient who was previously healthy. One week previously, he turned the temperature of the electric blanket to the highest setting and maintained it for 24 h, then he developed nausea, vomiting, brown urine, general weakness, and muscle pain. No specific treatment was given. None of his family members had experienced similar symptoms. On the day of admission, the patient’s symptoms worsened, and he also developed cough, dyspnea, edema, palpitations, and chest tightness. Physical examination showed: temperature 36.2°C; heart rate 93 beats/min; respiration 24 beats/min; blood pressure 163/100 mmHg; poor mental status; respiratory sounds of both lungs were slightly lower, wet rhonchi could be heard in the left lower lung; lower limb edema; muscle strength of both upper limbs 3/5; and muscle strength of both lower limbs 2/5. The remaining physical examination was normal.

Upon admission, his blood analysis showed that white blood cell count was 14.91 × 10^9^/L (normal range: 3.5 × 10^9^–9.5 × 10^9^/L), hypersensitive C-reactive protein level was 52.44 mg/L (normal range: <5.2 mg/L), and neutrophil percentage was 89.5% (normal range: 40–75%). These results showed that the patient had a systemic inflammatory reaction. To clarify the cause of inflammation, we tested him for endotoxin, (1–3)-β-D-glucan, BB virus DNA, cytomegalovirus DNA, SARS-CoV-2 DNA, sputum and urine bacterial culture, and routine stool examination; all of which were negative. Only the level of anti-streptolysin O (ASO) increased slightly: 144 IU/mL (normal range: 0–116 IU/mL). Therefore, we thought that his inflammation may have been related to streptococcal infection. Blood laboratory tests (Table 1) showed that he had acute kidney injury (AKI), hyperkalemia, hypercoagulopathy, and hyper-creatine-kinase-emia. The clinical manifestations of myoglobinuria, myalgia, myasthenia, and hyper-creatine-kinase-emia, and a history of exposure to a high-temperature environment, led us to consider that the patient had acute RM caused by heat stroke. His routine 12-lead electrocardiogram showed ventricular rhythm and peaked T waves, which we considered to be associated with hyperkalemia. Arterial blood gas analysis was suggestive of metabolic acidosis. Electromyogram showed myogenic damage to his limbs, with more severe damage in the lower extremities and proximal limbs. The results of his autoantibody profiles (Table 2), thyroid function, thoracic, abdominal and cardiac computed tomography, cardiac color Doppler ultrasonography and left ventricular systolic function measurements, and cranial magnetic resonance imaging were normal.

**Table 1:** Blood laboratory test results during the patient’s hospitalization.

Laboratory tests	Day 1	Day 15	Day 30	Normal reach
CK (U/L)	82,435	2,237	183	50–310
Mb (ng/mL)	>3,769	>3,769	564.77	<105.7
SCr (μmol/L)	503	198	155	59–104
UREA (mmol/L)	36.65	19.56	11.94	2.6–9.5
UA (μmol/L)	1,105	391	529	200–415
AST (U/L)	4,134	104	27	15–40
ALT (U/L)	1,140	48	18	9–50
LDH (U/L)	>10,000	2,249	431	120–250
BNP (pg/mL)	140.68	75.13	43.12	<100
α-HBDH (U/L)	>10,000	2,356	396	72–182
Potassium (mmol/L)	6.99	4.69	4.02	3.5–5.3
TG (mmol/L)	2.99	1.73	1.34	<2.26
WBC (10^9^/L)	14.91	10.12	7.83	3.5–9.5
NEUT%	89.5	82.3	62.4	40–75
Hs-CRP (mg/L)	52.44	16.24	10.31	<5.2 mg/L
D2 polymers (μg/mL)	2.69	4.29	1.86	0.0–0.5
ASO (IU/mL)	144	None	None	0–116

CK, Creatine kinase; Mb, Myoglobin; SCr, Serum creatinine; UA, Uric acid; AST, Aspartate aminotransferase; ALT, Alanine aminotransferase; LDH, Lactate dehydrogenase; BNP, Brain natriuretic peptide; α-HBDH, α-Hbdl reagent; TG, Triglyceride; WBC, White blood cell count; NEUT%, Neutrophils percentage; Hs-CRP, Hypersensitive C-reactive protein; and ASO, Antistreptolysin O.

**Table 2:** Examination of myositis spectrum and autoantibody spectrum of patients.

Autoantibodies	Result
Anti-HMGCR antibody	Negative
Anti-cN1A antibody	Negative
Anti-MDA-5 antibody	Negative
Anti-OJ antibody	Negative
Anti-KS antibody	Negative
Anti-ZO antibody	Negative
Anti-HA antibody	Negative
Anti-SCL-70 antibody	Negative
Anti-PM-SCL100 antibody	Negative
Anti-PM-SCL75 antibody	Negative
Anti-KU antibody	Negative
Anti-KU antibody	Negative
Anti-Th/To antibody	Negative
Anti-Fibrillarin antibody	Negative
Anti-NOR-90 antibody	Negative
Anti-JO-1antibody	Negative
Anti-PL-7antibody	Negative
Anti-PL-12antibody	Negative
Anti-EJ antibody	Negative
Anti-SRP antibody	Negative
Anti-Mi-2antibody	Negative
Anti-TIF1γantibody	Negative
Anti-Ro-52 antibody	Negative
Anti-SAE1 antibody	Negative
Anti-SAE2 antibody	Negative
Anti-NXP2 antibody	Negative
Anti-nRNP/Sm antibody	Negative
Anti-Sm antibody	Negative
Anti-PCNA antibody	Negative
Anti-SS-B antibody	Negative
Anti-SS-A antibody	Negative
Anti CENP-B antibody	Negative
Anti-dsDNA antibody	Negative
Anti-nucleosome antibody	Negative
Anti-histone antibody	Negative
Anti-ribosomal P protein antibody	Negative
Anti-mitochondrial antibody-M2	Negative

**Figure 1:**
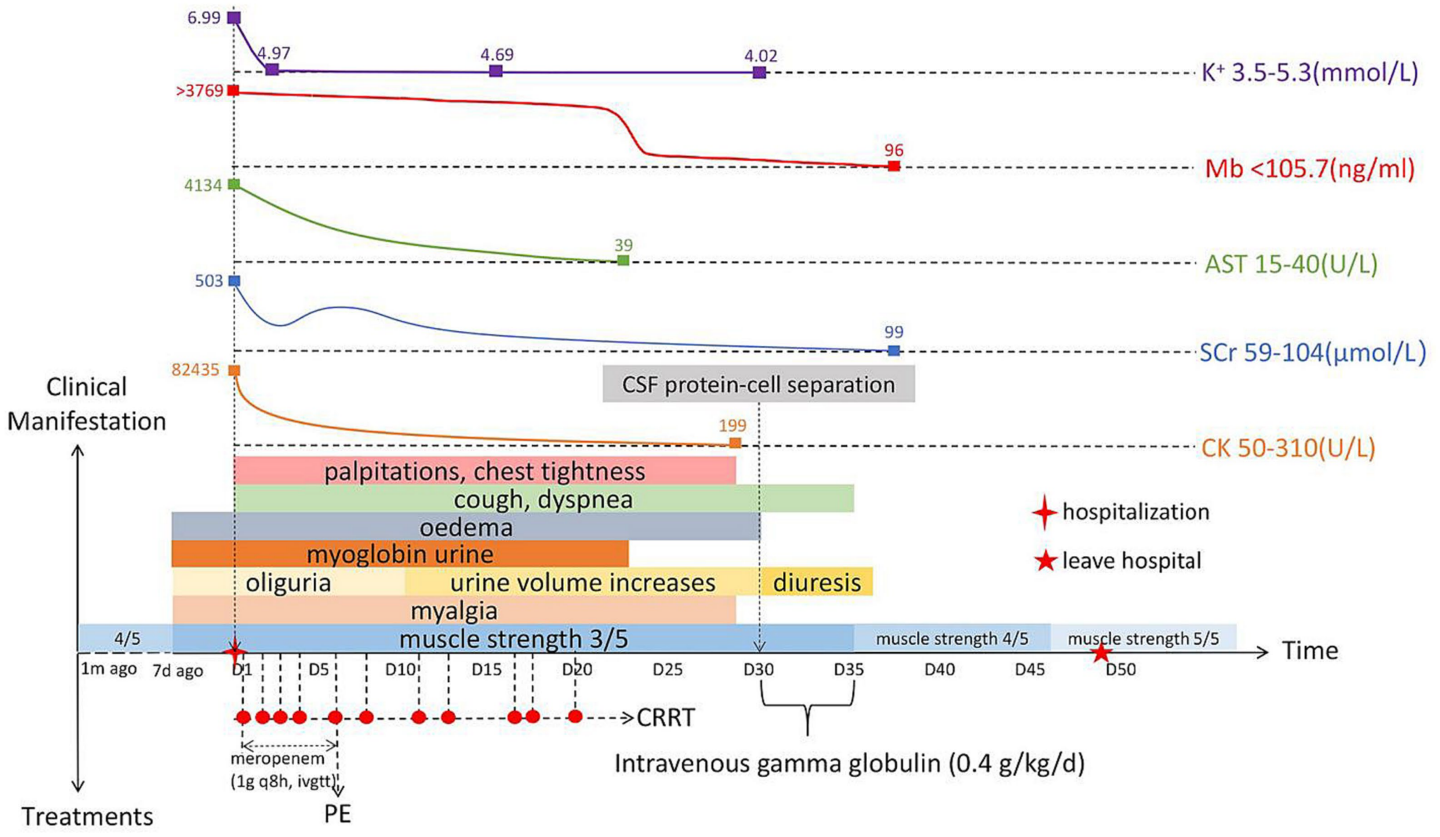
Timeline of the patient’s clinical manifestations, treatments, and laboratory examinations. K+, Blood potassium; Mb, Myoglobin; AST, Aspartate aminotransferase; SCr, Serum creatinine; CK, Creatine kinase; CRRT, Continuous renal replacement therapy; CSF, Cerebrospinal fluid; q8h, Every 8 h; ivgtt, Intravenous guttae; and PE, plasma exchange.

We used normal saline for fluid resuscitation; sodium bicarbonate for acidosis; calcium gluconate for hyperkalemia; beta-blocker to decrease blood pressure and normalize ventricular rate; meropenem (1 g every 8 h, intravenous guttae) as anti-infective treatment for 7 days; 5,000 IU low molecular weight heparin for anticoagulation; parenteral nutrition support; one plasma exchange (PE); and 11 sessions of bedside continuous renal replacement therapy. After the above treatment, most of the blood laboratory tests returned to normal on day 30 (Table 1). All organ functions were restored, and the 24-h urine output gradually increased and entered the polyuric phase. The patient did not excrete tea-colored urine, and the symptoms of muscle pain, nausea, and vomiting improved (Figure 1).

Although the laboratory test results recovered well, the symptoms of weakness in both lower limbs did not improve, and muscle strength was always 3/5. We invited the Department of Neurology to hold a consultation, and they recommended completing the inspection of the 26-item antimyositis antibody profile (Table 2), urinary organic acid, genetic metabolic disease amino acid, and acylcarnitine profiles to judge whether the patient had myopathy. However, all the above-mentioned tests were negative. ASO level was elevated and erythrocyte sedimentation rate increased. Therefore, the possibility of neurological infection could not be ruled out. We completed lumbar puncture on the day 30, cerebrospinal fluid (CSF) analysis showed that white blood cell count was 1 × 10^6^/L (normal range: 0–8 × 10^6^/L), and pathogens were not detected in CSF culture. However, CSF albumin was 824.0 mg/L (normal range: 100–300 mg/L) and total CSF protein 1413.0 mg/L (normal range: 150–450 mg/L). These results showed that the patient had the phenomenon of protein–cell separation in CSF. We inquired again about the patient’s medical history. He disclosed that his lower limbs had been weak 1 month before he was admitted to hospital but it did not affect his walking. He did not think it was important, so he did not tell us this key information before. We estimated that from 1 month to 7 days before admission, his lower limb muscle strength may have been 4/5. Finally, he was diagnosed with GBS by the Department of Neurology. After intravenous gamma globulin (0.4 g/kg/day) was administered for 5 days, the limb muscle strength recovered to 4/5, and to 5/5 at the time of discharge from hospital (Figure 1). He was followed up for 1 year and was recovering well.

## 3 Discussion

Rhabdomyolysis has been reported to occur in about a third of hospitalized patients with heatstroke (5), and the causes of heatstroke often include poor heat-dissipation mechanisms, strenuous exercise, and high environmental temperatures, and this disease usually occurs in hot weather (6). Overheating of electric blankets is a rare form of heatstroke that occurs in winter, and by reviewing the literature, we found only three patients with RM due to the use of electric blankets in winter; two patients died (7) and one survived (8). These remind us that heatstroke may also occur in cold seasons, which can induce RM.

We found seven detailed reports of RM complicated with GBS by reviewing the literature (Table 3), which makes eight cases in total including the present case. All eight patients were male, which means that men are more likely to suffer from RM complicated with GBS. Six patients had precursor events (four infections, one vaccination, and one pyrexia). The proportion of male patients and patients with precursor infections was higher among GBS patients with elevated serum creatine kinase (CK) levels (15), which is in line with our results.

**Table 3:** Literature review and patient data.

		Case 1	Case 2	Case 3	Case 4	Case 5	Case 6	Case 7
Clinical manifestation	Gender	Male	Male	Male	Male	Male	Male	Male
	Age, years	25	54	24	29	21	3	12
	Precursor event	-	-	Fever	Fever	Diarrhea	Meningococcal	Diarrhea
							Vaccination	
	Limb weakness	Arms and Legs 0/5	Legs 2/5	Arms 3/5; Legs 2/5	Legs 2/5	Arms and Legs 0/5	Proximal extremity 2/5	Proximal extremity 3/5
							Distal extremity 1/5	Distal extremity 2/5
	Myalgia	Double upper limbs	Lower limbs and back	None	Double lower limb	Lower limbs and lower back	Double lower limb	None
	Nerve root pain	√	None	None	None	None	√	√
	Dyspnea	None	√	√	None	None	√	√
Laboratory tests	CK max (U/L)	10,150	1,134	7,002	24,240	1,917	7,908	17,840
	Mb max (ng/mL)	Not detected	-	>1,000	-	580	>1,200	491
	AST max (IU/L)	-	-	274	549	-	388	-
	ALT max (IU/L)	-	-	476	220	-	261.2	97.5
	SCr max (μmol/L)	484	-	397.8	Normal	-	Normal	169.6
	Serum electrolyte	Normal	-	K↑P↑Ca↓/Metabolic Acidosis	-	Normal	Normal	Normal
	Blood gas analysis	-	Respiratory failure	Respiratory failure	-	-	Normal	Respiratory failure
	Etiological examination	-	-	Unknown	Cytomegalovirus infection	*Campylobacter Jejuni* infection	None	Unknown
	Serum AGAs	-	Normal	Normal	-	GM1-IgM(+)	normal	GM1-IgG(+)
						GD1b-IgM(+)		GD1b-IgG(+)
	CSF protein-Cell separation	Negative	Negative	Positive	Positive	Positive	Positive	Positive
	Electroencephalogram	AMSAN	AIDP	AMSAN	-	AMAN	AMAN	AMSAN
	Muscle biopsy	Necrosis and edema	-	Normal	Necrosis	-	-	-
	Cranial and spinal MRI	-	-	-	-	-	Normal	Normal
Treatments	RRT	-	-	-	-	-	-	-
	PE	√	√	-	√	√	-	√
	PD	-	-	√	-	-	-	-
	Gamma globulin	-	-	-	-	√	√	√
	Glucocorticoid	-	-	-	√	-	√	-
	Mechanical Ventilation	-	√	√	-	-	-	√
Time/Final result		m10/Healed	d35/Double lower limb 4/5	d4/Died	d10/Double lower limb 4/5	m3/Healed	m4/Healed	m5/Healed
Cite		(9)	(10)	(11)	(12)	(13)	(14)	(14)

√: present; −: unreported; CK, Creatine kinase; Mb, Myoglobin; AST, Aspartate aminotransferase; SCr, Serum creatinine; AMAN, Acute motor axonal neuropathy; AIDP, Acute inflammatory demyelinating polyneuropathies; AMSAN, Acute motor and sensory axonal neuropathy; RRT, Renal replacement therapy; PE, Plasma exchange; PD, Peritoneal dialysis; AGAs, Anti-ganglioside antibody; and CSF, Cerebrospinal fluid.

It is reported that streptococcal infection can be a precursor event of RM (16). Our patient’s ASO level only increased slightly, and no pathogen was cultured in the blood, which is inconsistent with the severity of RM. Therefore, in our case, the cause of RM was more likely to have been heatstroke. Almost 72% of GBS patients have previous infections (17), and streptococcal infection can also be associated with GBS (18). ASO was the only positive test in our patient. However, because no pathogens were cultured from his blood, sputum and CSF, the onset time of GBS was 1 month ago. Therefore, we can only speculate that his GBS may have been related to streptococcal infection.

Both RM and GBS can have clinical manifestations of limb weakness. Eight cases (100%) patients all developed different degrees of limb weakness. All of them had ≤2/5 muscle strength in both lower limbs and were unable to walk. This makes it difficult for us to determine whether they had GBS in the first place, which means that it is easy to miss the diagnosis in the clinic. However, we found that the symptoms of bilateral lower limbs weakness in cases 3, 5, 6, and 7 and our patient all appeared before the CK elevation, indicating that the decrease of muscle strength in these five patients is caused by GBS. This shows that we can consider whether RM patients are complicated with GBS by judging the time sequence of limb weakness and CK elevation.

Seven patients, including ours, showed pain in different regions: four with muscle pain, one with neurogenic pain, and two with combined muscle and neurogenic pain. One of the triad of RM is muscle pain. About 34.5% of GBS patients have pain symptoms, and neuropathic pain and muscle pain are the most common types of pain in GBS patients (19). This means that when RM patients have only muscle pain, it is difficult to determine whether the patient had GBS at first, which may lead to missed diagnosis. Therefore, when RM patients have pain, we need to carefully determine the nature of their pain, especially when the patient has nerve root pain, it is necessary for us to conduct further examination to confirm the presence of GBS.

Rhabdomyolysis is often accompanied by elevated serum aminotransferase levels. However, most of these increases are not from liver cell damage, and are more likely to be derived from skeletal muscle injury (20). Five patients showed varying degrees of elevated serum aminotransferase levels. All of them had severe RM (CK >5,000 U/L) but none had prior liver disease. Therefore, for patients with no previous history of liver disease and decreased muscle strength, if they also have elevated serum aminotransferase levels, we should consider whether they have liver damage or RM.

Five patients, including ours, had dyspnea, and three of them received mechanical ventilation due to respiratory failure. It is reported that about 30% of GBS patients have respiratory failure, and the main mechanism involves respiratory myasthenia, autonomic nerve involvement, and progressive bulbar palsy (21). Few patients with RM have respiratory muscle paralysis that evolves into respiratory failure (22). Therefore, when an RM patient has dyspnea or even respiratory failure caused by respiratory muscle paralysis, we should consider whether they have GBS.

Acute kidney injury is one of the serious complications of RM, with a reported incidence of 13–50%. Its pathological basis is acute tubular necrosis, and its mechanism may be related to renal vasoconstriction, tubular obstruction, and direct cytotoxicity of myoglobin (23). When RM patients are complicated with AKI, a lot of muscle cell contents dissolve into the blood, which makes electrolyte disorder and metabolic acidosis more serious. Four patients, including ours, presented with AKI. However, GBS patients rarely show an increase in serum creatinine. GBS and RM can be caused by infection (16–18), and the clinical manifestations of these two diseases are similar; therefore, it is easy to miss diagnosis. Although AKI is rare in GBS, we think that when it does occur, it is necessary to carry out further muscle enzymatic examination to determine whether the patient is complicated with RM.

Rhabdomyolysis complicated with GBS should be differentiated from diseases such as hereditary myopathies, metabolic myopathies, inflammatory myositis, and myopathies due to sepsis. Muscle biopsy, genetic or serological tests are helpful to make a definite diagnosis. In the literature, three of seven patients underwent muscle biopsy (two cases of muscle necrosis and edema, and one normal case). In our case, to rule out hereditary and metabolic myopathy, we measured the autoantibody, amino acid and acyl carnitine spectrum of hereditary, and metabolic myopathy, but did not find anything unusual. After treatment, CK level returned to normal, so we did not conduct muscle biopsy.

The management of RM is aimed at preventing AKI, and the mechanism of AKI in RM is related to urinary myoglobin. Therefore, the *in vitro* removal of myoglobin from the blood may be an effective method of prevention and treatment (24), such as with PE and renal replacement therapy (25). Intravenous gamma globulin and PE have been shown to be effective treatments for GBS (26). Therefore, PE may be a necessary and effective treatment for patients with RM complicated with GBS. Unfortunately, PE was not used in the only fatal case among the seven in the literature. Including our patient, six underwent PE, and all these patients recovered and were discharged successfully.

## 4 Conclusion

Rhabdomyolysis caused by overheating of electric blankets reminds us that heatstroke can also occur in cold winters. RM and GBS have some similar symptoms, such as muscle pain and muscle weakness. This means that when RM patients are complicated with GBS, it is easy to cause missed diagnosis at an early stage. By reviewing the literature, we found that we can consider whether RM patients are complicated with GBS by judging the time sequence of limb weakness and CK elevation. In addition, if RM patients have clinical manifestations of respiratory muscle paralysis and nerve root pain, we can make an electroneurogram or CSF puncture to help us judge whether he is complicated with GBS. If GBS patients’ serum aminotransferase and serum creatinine increasing significantly, we can conduct muscle enzyme or muscle biopsy to help us judge whether he is complicated with RM or other neuromuscular diseases. When RM is complicated with GBS, PE should be the first choice of treatment, and it can achieve a better prognosis.

## Data availability statement

The original contributions presented in the study are included in the article/supplementary material, further inquiries can be directed to the corresponding authors.

## Ethics statement

The studies involving humans were approved by Ethics Committee of Affiliated Traditional Chinese Medicine Hospital of Southwest Medical. The studies were conducted in accordance with the local legislation and institutional requirements. Written informed consent for participation was not required from the participants or the participants’ legal guardians/next of kin in accordance with the national legislation and institutional requirements. Written informed consent was obtained from the individual(s) for the publication of any potentially identifiable images or data included in this article.

## Author contributions

DJ: Writing – original draft. MZ: Data curation, Visualization, Writing – original draft. QH: Supervision, Writing – review & editing. QZ: Supervision, Writing – review & editing. XL: Project administration, Writing – review & editing.
